# Snakebite: When the Human Touch Becomes a Bad Touch

**DOI:** 10.3390/toxins10040170

**Published:** 2018-04-21

**Authors:** Bryan G. Fry

**Affiliations:** Venom Evolution Lab, School of Biological Sciences, University of Queensland, St. Lucia, QLD 4072, Australia; bgfry@uq.edu.au; Tel.: +61-4-0049-3182

**Keywords:** snakebite, envenomation, venom, antivenom

## Abstract

Many issues and complications in treating snakebite are a result of poor human social, economic and clinical intervention and management. As such, there is scope for significant improvements for reducing incidence and increasing patient outcomes. Snakes do not target humans as prey, but as our dwellings and farms expand ever farther and climate change increases snake activity periods, accidental encounters with snakes seeking water and prey increase drastically. Despite its long history, the snakebite crisis is neglected, ignored, underestimated and fundamentally misunderstood. Tens of thousands of lives are lost to snakebites each year and hundreds of thousands of people will survive with some form of permanent damage and reduced work capacity. These numbers are well recognized as being gross underestimations due to poor to non-existent record keeping in some of the most affected areas. These underestimations complicate achieving the proper recognition of snakebite’s socioeconomic impact and thus securing foreign aid to help alleviate this global crisis. Antivenoms are expensive and hospitals are few and far between, leaving people to seek help from traditional healers or use other forms of ineffective treatment. In some cases, cheaper, inappropriately manufactured antivenom from other regions is used despite no evidence for their efficacy, with often robust data demonstrating they are woefully ineffective in neutralizing many venoms for which they are marketed for. Inappropriate first-aid and treatments include cutting the wound, tourniquets, electrical shock, immersion in ice water, and use of ineffective herbal remedies by traditional healers. Even in the developed world, there are fundamental controversies including fasciotomy, pressure bandages, antivenom dosage, premedication such as adrenalin, and lack of antivenom for exotic snakebites in the pet trade. This review explores the myriad of human-origin factors that influence the trajectory of global snakebite causes and treatment failures and illustrate that snakebite is as much a sociological and economic problem as it is a medical one. Reducing the incidence and frequency of such controllable factors are therefore realistic targets to help alleviate the global snakebite burden as incremental improvements across several areas will have a strong cumulative effect.

## 1. Epidemiology

Snakebite is the most neglected of all tropical diseases and requires global solutions [1,2,3,4,5,6,7,8]. Indeed, it is only very recently that snakebite was added back onto the WHO (World Health Organization) list of neglected tropical diseases, after being removed in 2013 [9]. Geographically, the greatest impact of snakebite is in the tropical and subtropical regions (with India considered as having the highest rates of incidence and mortality), due to a combination of factors ranging from snake density to developing world status resulting in many local people not having access to footwear, flashlights or adequate medical care [8,10,11,12,13]. Estimates for snakebite incidence vary widely, ranging from 1.8 million to 5.4 million bites globally per year with tremendous socio-economic impacts [10,11,12,13,14,15,16,17,18,19]. However, the lack of robust record keeping in the most affected regions, combined with many victims not presenting to hospital due to logistical or cultural reasons, lead to estimates widely-recognized as likely being gross underestimations. The lack of robust incidence and mortality rates for snakebite contributes to this being a neglected public health issue as the true clinical and socio-economic impact is not fully recognized [13]. Therefore, only the tip of the iceberg is seen in regards to the medical, social and economic impacts of snakebites. Consequently, snakebite is virtually excluded from foreign aid conversations, with funding instead targeting higher profile tropical diseases such as HIV/AIDS and malaria. This is despite snakebite being a socially destabilizing force that has a very high medical and social value-for-money in regards to the economy of treatment relative to the more expensive treatment for chronic diseases [3]. Thus, the limited understanding of snakebite epidemiology has relegated snakebite to an neglected tropical disease status despite the social and economic catastrophic impact it may have [19]. Entire family groups may be plunged into poverty if the person killed or suffering permanent disability is the primary source of income. Semantics plays a further influence as snakebite is not an infectious disease, which has contributed to its neglect as it is not usually viewed as a “typical” neglected tropical disease. 

## 2. Human Influences on Snake Movements and Behavior

### 2.1. Increase in Snake Numbers, Activity Periods and Distributions due to Human Activities

While a certain level of human/snake accidents is inevitable, economic displacement, habitat reshaping and climate change have increased the level of interactions and thus the incidence of snakebites. A contributing issue is the increased poverty in many regions due to global economic trends that especially affect poor settlers in tropical countries, with a major variable being the poor condition of many housings which enable snakes to get into homes [10]. Farming practices in rural areas in some cases have increased snake density as have the development of slums [13]. The species benefiting from such modified habitats typically are rodent feeding, habitat generalists, such as *Daboia* in Indian rice fields, *Echis* in Nigerian farms, *Naja* in Indian slums, *Oxyuranus* in Papua New Guinea sugar cane plantations, and *Pseudonaja* in Australian farming regions. Climate change is extending the annual activity periods of snakes in addition to increasing the geographical ranges of some species [20,21,22,23,24,25]. In addition, the increase of tropical storms also contributes to the rise in snakebites, with snakebites the second cause of fatalities after flooding [26]. Such natural disasters also affect the access of humans to health centers (overflood rivers for example), or destroy health facilities, making access to healthcare more difficult.

### 2.2. Snake Repellants

Typically, snake repellants are mixtures of various chemicals, some of which may have severe effects on any vertebrate life including humans and their pets [27,28] and none of which have been shown to have a snake-specific repellant effect [29]. Supporting evidence has been lacking and generally limited to testimonials of dubious credibility [30]. Testing in controlled laboratory conditions revealed their inefficiency [31]. The use of snake repellants with dubious efficacy may simply give people a false sense of security and thus less likely to take prudent steps to reduce snakes around the house such as keeping vegetation cut back and removing (or raising up) items under which snakes can shelter.

### 2.3. Translocations

While any activity that reduces human/snake accidental encounters may be beneficial, snake removal and relocation is not a straightforward issue. Short-distance translocations within the estimated normal home range of the species is desirable from the perspective of the snake [32,33] but undesirable from the human perspective as the snake is likely to return or be encountered again [32,33,34,35]. For large, active species such as king cobras (*Ophiophagus hannah*), the home range may be as large as 6 km^2^ while, for smaller species, such as arboreal vipers, the ranges may be as small as 0.01 km^2^ [36,37]. It has been shown that long distance translocation outside of the home range of the snakes results in behavioral patterns consistent with distress and such snakes have very poor outcomes including high mortality rates [35,38,39,40,41,42]. Thus, in addition to animal ethics considerations, distressed snakes that are exhibiting atypical behaviors may be encountered in highly agitated states and therefore may react more defensively and envenomate more readily than snakes that have not been translocated. Long distance translocation as a contributing variable to snakebite has not been investigated in this context but must be the focus of future behavioral research. 

## 3. Inappropriate First-Aid and Treatment

Fatality rates of venomous snakebites are much lower than popularly viewed, due to high rates of “dry bite” (no venom injected) or sublethal amounts injected, with over half the cases displaying little or no symptoms and this rate may reach up to 80% for some short fanged genera such as *Pseudonaja* [11,43,44,45,46]. Other variables include the misattribution of bites from harmless (non-venomous species or non-lethal rear-fanged venomous species) snakes as being from highly venomous species. Thus, a typical person being bitten by a snake has a high chance of surviving the bite untreated. Therefore, the ineffective remedies discussed below gain false support for their efficacy. In some cases, the victims survive despite radical alternative treatments ranging from drinking poisons thought to neutralize the effects of venom to inflicting deep wounds upon themselves in a vain attempt to bleed the venom out. The critical examinations in the subsections below reveal not only the lack of evidence-based support for such alternative first-aid and treatment options, but also clearly demonstrates that in some cases they actually worsen the outcomes. 

While pressure-immobilization is a first-aid technique supported by data as being effective for bites from certain snake species [47] and antivenom the only validated specific treatment [48,49], a number of alternative methods [50] have been advocated throughout history and continuing into the modern age [51]. While none of these are supported by evidence-based research, their popularity persists for a myriad of cultural reasons ranging from superstition/religion to the unavailability of antivenom due to supply or economic reasons leading people to seek out alternative treatments out of sheer desperation [52,53,54,55]. Such inappropriate first aid methods are not confined to traditional healers, with one survey revealing doctors advocating tourniquets (33%), snakestones (12.8%), ice (22.5%), incision and application of herbs (5%), suction (11.8%), or electric shock (1.6%) [8].

### 3.1. Traditional Healers 

The lack of regional medical care in many rural parts of the developing world results in local biases, whereby the majority of developing world snakebite victims will first seek out traditional healers rather than modern clinical care [13,17,55,56,57,58]. When modern medical care is finally sought after a delay due to traditional healers being first sought out [59], the elapsed time has worsened symptoms and thus increasing the likelihood of a poor outcome [60], and it is often modern medicine that is blamed rather than the fault rightfully directed at the delay and the practices of traditional healers which in many cases actually contribute to the poor outcomes [14,52,61,62]. Traditional medicine is thus a major problem and cultural intransience makes engagement with traditional healers a particularly challenging sociological problem to deal with. As they will remain the primary source for medical advice, this necessitates a dialog strategy rather than just direct disqualification [17].

### 3.2. Mechanical First-Aid

#### 3.2.1. Cutting 

Cutting the bite site to promote local bleeding in the misguided hope that this will remove the venom from the body is the oldest form of attempted treatment [63]. Such inappropriate cuttings have immediate dangers ranging from the severing of tissues including arteries, nerve bundles and tendons. In addition, due to the use of non-sterile implements, including chewing on the wound, the chances of secondary infection are very high [64,65,66,67,68]. In envenomations which result in net anticoagulant effects (whether through direct inhibition of the clotting cascade, pseudo-anticoagulant functions producing weak, readily friable fibrin clots, or procoagulant mechanism which in human victims consume clotting factors), such cutting may result in severe blood loss due to persistent bleeding as a consequence of the venom induced uncoagulable blood. 

#### 3.2.2. Tourniquet

The use of a tourniquet is also an old and still often practiced inappropriate form of first-aid, with up to 98% of patients in some developing countries presenting with tourniquets applied and is typically accompanied by wounding, often with catastrophic outcomes [26,69,70,71,72,73]. Lack of local oxygen supply will rapidly damage tissue, which if for a prolonged enough period, will result in tissue death and lead ultimately to amputation. Unfortunately, use of this damaging practice is extremely common, which may not retard the spread of venom but almost certainly will worsen local damage [72,74,75]. Further, when the tourniquet is released, there may be an abrupt systemic absorption of venom, rapidly resulting in severe systemic envenoming [76].

#### 3.2.3. Extraction 

“Black stones” (charred bone) are a long-advocated indigenous first aid treatment. The premise is that they will absorb the venom from the bite site due to the porous nature of the material. Despite no evidence supporting their use and scientific studies disproving efficacy, their use remains widespread [60,77,78,79].

Methods for attempted extraction of venom using negative pressure ranges from the use of mouth suction including so-called “venom extractors” of varying mechanical complexity. Such methods rely upon two premises being true: (a) that venom remains in the immediate vicinity of the bite site for a sufficient period that it is available for removal; and (b) that negative pressure will preferentially draw up venom injected into the tissue rather than closer to the surface blood or lymphatic fluids instead. However, studies have shown that venom rapidly diffuses away from the bite site [80,81], which is indeed consistent with its use as a rapidly acting predatory weapon. The use of the mouth is a low-pressure suction that is utterly ineffective. However, it includes the remote chance of the transmission of venom from the bite site to the person doing the sucking if an oral injury is present (e.g., damaged gums) and thus in theory may result in envenomation of the person doing the sucking due to venom on the skin. Further, the transmission of oral bacteria to the bite site may result in a secondary infection [64]. Suppliers of mechanical extractors invariably make grandiose statements regarding their efficacy (cf. [82,83]). 

The conspicuous lack of independently reviewed data has resulted in their wide-spread criticism [84,85,86]. In contrast, a scientific study that examined the performance of a mechanical extractor using human volunteers, reported less than 2% of the radiolabeled mock-venom was recovered by the extractor [87]. Other research using a pig model (chosen as pigs have similar skin anatomy to humans) reported not only a lack of efficacy but also a conspicuous worsening of local effects due to skin damage from the extractors themselves resulting in lesions [88,89]. Thus, as venom is not injected superficially, suction is not able to remove venom injected deep into the tissues.

### 3.3. Freezing, Burning and Shocking

The use of extreme cold as a first-aid and treatment option is another method which has been shown to result in local tissue damage but without any benefit in regards to the envenomation itself [85,86,87,88,89,90,91,92,93,94]. In addition to hypothermic effects, the application of cold may hasten the spread of venom due to cold-triggered vasodilatory effects. Conversely, the use of intense heat has been a parallel long-standing attempt at neutralizing the venom, with methods ranging from gunpowder to heated metal implements. Chemical burning has also been attempted through the use of caustics such as nitric acid, potassium hydrate and silver nitrate [90,95]. 

The use of electricity has gained recent (but scientifically unsupported) favor (with lethal outcomes from electrical shock in some cases). Venom proteins do not differ in amino acid biochemistry from other proteins, including those in the human body [96]. Thus, electricity would not preferentially neutralize venom proteins and any voltage sufficient to damage venom proteins would equally damage the body proteins of the victim. The suppliers of such electrical devices, similar to the sellers of mechanical suction devices, have made outlandish and evidence-free statements [97,98,99,100,101,102,103]. In contrast to the evidence-free advocation of the method, peer-reviewed and published research on the efficacy of electricity demonstrated a complete failure of the technique [99,104,105,106,107,108,109]. In 1990, the US Food and Drug Administration implemented a formal ban of electrical devices being promoted as having any therapeutic usefulness [99]. Despite this ban, webpages can still be readily accessed that recklessly promote such devices [110] despite these methods having been disproven in recent investigations [99,104,105,106,107,108,109] and the subject of a ban by the FDA [99]. In addition to the lack of efficacy, the method itself is dangerous as it may lead to burns, lethal heart attacks or electrocution [109,111,112]. In one particularly noteworthy case, a snakebite victim bitten in the face while free-handling his pet rattlesnake, had his neighbor attach a car spark plug wire to his lip and then rev the car engine for five minutes to generate maximum electrical output, during which period, the patient promptly defecated upon himself and rapidly lost consciousness while suffering severe burns and other electrocution effects [109]. Laboratory tests undertaken while he was in the hospital revealed the envenomation to have been a mild one. 

### 3.4. Chemical for First-Aid and Treatment

Ingestion of alcohol is amongst the most common of all folk-remedies for snakebite and other envenomations [113,114]. In addition to the potential for masking neurological symptoms, alcohol may actually worsen effects due to its vasodilatory and anticoagulant actions [115,116,117]. 

The ingestion of plant extracts and local application of poultices are other antiquated remedies that lack robust supporting evidence [65,118,119,120,121,122]. The majority of patients in some locations have been “treated” by plant extracts prior to presenting at a modern medical facility, but with no discernable benefit and a worsening of envenomation symptoms due to the time delay [121]. Laboratory experiments attempting to show therapeutic benefit use sets of conditions radically different than those used by traditional healers or even achievable in the real world, or with poor experimental designs including lack of relevant controls [121,123,124,125,126]. Conversely, many of these plant extracts are themselves extremely toxic, such as the strychnine containing monkey fruit [127]. Further, the application of poultices may result in secondary infection. 

Vitamin C (ascorbic acid) has long been a folk-remedy for a myriad of ailments [128] including snakebite [129,130,131]. As with other alternative first-aid/treatment options, there is a conspicuous lack of supporting evidence and no mechanism for its efficacy has been proposed or tested. 

## 4. Issues and Controversies in Modern Medical Care

### 4.1. Pressure Bandage First-Aid

Few areas are as contentious in modern medicine as the use (outside of Australia) of the pressure-bandage and immobilization first-aid technique developed by Struan Sutherland [47]. As venom typically travels via the lymphatic vessels (with intravenous envenomation being exceedingly rare), it was demonstrated that the application of a compression bandage followed by splinting of the affected limb greatly retarded the flow of venom and thus delayed the development of systemic effects. Australian venomous snakes rarely cause significant local swelling or tissue damage with the exception of *Demansia* (swelling) and *Pseudechis* (tissue damage) [132]. Instead, Australian elapid envenomations are characterized by lethal systemic effects including pre- and post-synaptic neurotoxicity and/or consumptive coagulopathy [132]. While the technique as applied under idealized circumstances is effective, it is often not applied correctly and thus the real world benefit varies considerably [133,134,135]. 

However, the use of pressure-bandage and immobilization first-aid is particularly controversial in North America due to the perception that it may worsen localized tissue damage. Based upon this assumption, its use in American crotalids has been specifically discouraged [136,137]. The prevailing logic is that it is better to let the venom diffuse to reduce local tissue damage, with the trade off being a worsening of systemic effects but to a manageable degree as most pitvipers from North America are unlikely to rapidly cause lethal systemic effects. Thus, a joint statement issued by the American College of Medical Toxicology, the American Academy of Clinical Toxicology, the American Association of Poison Control Centers, the European Association of Poison Control Centres, the International Society on Toxinology, and the Asia Pacific Association of Medical Toxicology in 2011 (ACMT-AACT-AAPCC-EAPCC-IST-APAMT) read: “Given that the primary toxic effect of envenomation is local tissue injury, mortality is not an ideal outcome measure to extrapolate to human crotaline envenomation. Available evidence fails to establish the efficacy of pressure immobilization in humans, but does indicate the possibility of serious adverse events arising from its use. The use of pressure immobilization for the prehospital treatment of North American Crotalinae envenomation is not recommended.” [136,137]. Justification for this position was a single study using *Crotalus atrox* venom that showed a raised intracompartmental pressure in an animal model [138]. However, this same study showed a delay in mortality and also noted less swelling in the animals with pressure-immobilization. The study, however, was unable to address the other key issue of local necrosis. 

Considering the fundamental medical importance of proper first-aid, remarkably few other investigations have been carried out. The early work by Sutherland included studies on the effects with pitviper venoms such as *Crotalus adamanteus* and did show a delay in the development of systemic effects and yet with less local swelling [139]. These results are congruent with the above *C. atrox* study [138] as well as research by another group that showed a delay in *C. adamanteus* lethal effects [140] and another that showed delay in venom absorption but without an increase in swelling [141]. None of these laboratory-based studies reported upon the relative necrotic effects, likely due to the short-time periods for which the studies were conducted. However, in a study on *C. atrox* venom that left pressure-bandages on for 24 h, the experimental group with bandages had greater survivability and much less necrosis than the widespread tissue necrosis seen in the control group [142]. 

Studies on the use of pressure-bandage first-aid for *Daboia siamensis* envenomations in patients from Myanmar showed a reduction in systemic effects while conversely not recording an increase in local tissue effects [143]. Laboratory work with radiolabeled *D. siamensis* venom confirmed the delay in its systemic spread [144]. Consistent with the above, some authors have supported the use of pressure-immobilization for potently neurotoxic or coagulotoxic non-Australian snakes such as *Daboia*, *Dispholidus*, *Echis*, *Thelotornis Dendroaspis*, *Micrurus* and *Naja* [145,146]. 

Clearly more research urgently needs to be done on this critical aspect of first-aid as there is a disjunction between the theory and the body of literature in regards to the potential for necrosis being worsened by pressure-bandages [142]. The other feature of concern, the rise of intracompartmental pressure is also data deficient as one study has reported an increase in intracompartmental pressure [138] while another did not report an increase in swelling [141]. Further research will allow for evidence-based recommendations to be made as there is currently insufficient evidence to support or discourage the use of pressure bandages in North American crotalids

### 4.2. Fasciotomy 

Inappropriate cutting is not confined to the developing world or the private sector but extends into the halls of modern medicine in the form of fasciotomies [145]. This is the surgical attempt to alleviate intracompartmental pressure [147,148], a technique entirely appropriate in crushing injuries that produce extreme swelling and dramatic rises in intracompartmental pressure [149]. Medical practitioners unfamiliar with snakebite may view swelling as needing fasciotomies to reduce compartmental pressure due to their lack of the knowledge necessary to discern that the pathology of snakebite-induced subdermal swelling is radically different from the deep tissue swelling produced by traumatic injuries [150,151,152,153,154,155]. However, typically, the decision to send the patient to surgery for a fasciotomy is taken without the advisory tests to measure the intracompartmental pressure. In the absence of evidence of critical pressure rises within the affected limb, such radical surgery is entirely unjustified.

The official WHO guidelines for the treatment of snakebite state “The most reliable test is to measure intracompartmental pressure directly through a cannula introduced into the compartment and connected to a pressure transducer or manometer. In orthopaedic practice, intracompartmental pressures exceeding 40 mmHg (less in children) may carry a risk of ischaemic necrosis (e.g., Volkmann’s ischaemia or anterior tibial compartment syndrome). However, fasciotomy should not be contemplated until haemostatic abnormalities have been corrected, otherwise the patient may bleed to death. Animal studies have suggested that muscle sufficiently envenomed and swollen to cause intracompartmental syndromes, may already be irreversibly damaged by the direct effects of the venom. Early treatment with antivenom remains the best way of preventing irreversible muscle damage.” [156].

### 4.3. Antivenom 

#### 4.3.1. Antivenom Development, Supply and Distribution

The economics of antivenom production makes it a low-profit product for private pharmaceutical companies as it is the classic orphan drug: an expensive product with a limited shelf life and needed the most by those who can afford it the least [4,157,158]. Consequently, manufacturers of some very effective antivenoms are leaving the markets and neglect has led to avoidable deaths [159,160]. In some cases, such as sub-Saharan Africa, there are new antivenoms being developed [161,162] and, similarly, a new, cost-effective, antivenom is being developed for *Oxyuranus scutellatus* envenomations in Papua New Guinea [163,164]. 

However, even for countries with an adequate supply of effective antivenoms, their distribution may be concentrated in major urban cities with low snakebite incidence rather than the rural areas that suffer the highest rates of envenomations [165,166,167]. A particularly complicated legal and medical arena is the supply of antivenom for and treatment of exotic snakebites in zoological or private collections, which may be unattainable within a reasonable period and the attending doctors are likely to lack the experience necessary to treat such an emergency [168,169,170,171,172,173,174]. 

A significant complication is one of antivenom stability, with liquid-based antivenoms requiring continuous refrigeration, and thus research efforts are also investigating the stability of antivenoms at room temperature [175,176,177,178,179]. Investigations are also being undertaken for the development of antivenoms using new methodologies including monoclonal and recombinant antibodies, precipitations methods and immunoglobulin type [48,180,181,182,183,184,185]. 

Alternative treatments are not limited to the developing world as bites from snakes in exotic pet collections [186] pose unique legal and medical complications [48]. Compounding factors include lack of reliable information amongst private keepers regarding the venom effects of their species (typically reliant upon internet sources of dubious accuracy), validated emergency protocols and a generalized lack of private supply of exotic antivenoms due to cost, legal and supply issues [187,188]. This is particularly the case if the species is being kept illegally, which may cause the victim to avoid presenting to the hospital in a timely manner due to a fear of losing their collection and facing criminal charges. Thus, the problem of exotic snakebites is compounded by legislative prohibitions upon private keeping of venomous animals, which may simply drive the keeping underground and strangle the dissemination of knowledge regarding proper husbandry techniques, envenomation protocols and antivenom supplies, thereby exacerbating the situation.This situation may be alleviated by rational permit systems that require proper training and demonstrated competence, with suspension or loss of license for irresponsible actions such as posting pictures on Facebook of free-handling. Included in such a licensing system would be documentation of access to antivenom for the species being kept, whether through private procurement or as part of an antivenom depot. Such antivenom depots have been established in countries with such legislative foresight [168]. 

#### 4.3.2. Antivenom Adverse Reactions and Premedication

Adverse reactions to antivenom may be early or late developing due to the involvement of different pathological pathways, including early developing IgE anaphylaxis, early developing non-IgE-mediated anaphylactoid reactions, and the IgG and IgM mediated late developing adverse reaction of serum sickness [49,145,189,190,191,192,193,194,195,196,197,198]. However, premedication with adrenalin has been shown to significantly reduce the incidence of early adverse reactions [193,199,200,201,202,203]. One study resulted in the contrary conclusion that adrenalin was not effective since 2 out of 11 (18%) patients receiving adrenalin had allergic responses while 20 out of 86 (23%) not receiving any form of premedication developed allergic responses [204]. However, as noted by the authors themselves in this dissenting opinion, study limitations included the failure to accurately determine the antivenom infusion rates and the inability to determine duration of antivenom administration, both major variables in determining the relative correlation between premedication and adverse reaction rates. This dissenting opinion is also in contrast qualitatively with other studies: 2/66 (3%) of patients receiving adrenalin premedication developed a reaction to the antivenom, in contrast to 2/16 (12.5%) not receiving adrenalin as premedication who developed a reaction [205]; 5/65 (7.7%) adverse reaction rates in adrenalin premedicated patients versus 20/41 (28.3%) in patients without adrenalin premedication [199]; and adrenalin significantly reducing severe reactions to antivenom (by 43%) in a study of 1007 patients [193]. Thus, the weight of evidence in the available literature favors the use of adrenalin as antivenom premedication.

#### 4.3.3. Antivenom Dosage

As snake envenomation can cause permanent damage, early and aggressive antivenom treatment is linked to more favorable long-term outcomes than delaying treatment until severe symptoms appear [206]. The interaction of antivenom with venom is influenced by a wide range of variables, including the amount of venom delivered and the relative recognition of toxin isoforms within a particular antivenom. The WHO guidelines for antivenom production and control include results from “dose-finding studies”, in which several doses of antivenom are tested and the appropriate starting dose is selected based on the results [207]. Antivenom efficacy in a clinical setting is best ascertained if there is a readily observable physiological marker such as the rapid improvement in muscle tone in the case of post-synaptic nicotinic acetylcholine blocking toxins. 

In contrast, in venoms which cause consumptive coagulopathy, restoration of clotting factors may not be evident for 6–12 h or even longer [208]. Thus, in such envenomations, there is a data deficient period in which observable symptoms or physiological values do not provide indications of how well the venom has been neutralized by the antivenom. The absence of reliable markers has thus led to debates about how much antivenom should be given. An example based upon measurements of the concentration of antivenom bound venom in the plasma is the advocation of a one-size-fits-all-approach of one ampoule of Seqirus (formerly CSL) antivenom for Australian envenomations [206,209,210]. Critics of this approach argue that this ignores important variables such as the amount of venom injected and assumes that all bound venom is neutralized venom [211,212]. Measuring venom bound by antivenom in plasma does not equate to neutralization of the venom as the antivenom could be bound, for example, to a site distinct from functional sites thus the toxin may still be able to exert its pathophysiological action. In addition, studies that measure binding in plasma as a guide to antivenom dosing operate upon the assumption that toxins once bound stay bound. Therefore, as binding studies in the absence of testing for functional neutralization are only snap-shots in time, great caution must be used in interpreting such results into clinical practice (see Section 4.3.5). In addition, if the binding is not strong, then the antibody–venom protein complex may disassociate and the toxin resume exerting its action, with the antivenom thus acting as an intermediate reservoir for the toxins. Thus, in an optimal situation, even if all the venom is bound, there would be circulating free antivenom antibodies available to bind dissociated venom molecules before they are able to inflict their toxicity again. Therefore, a simplistic aim of achieving a 1:1 stoichiometry between antivenom and venom has several potential shortfalls.

Another fundamental dosing issue is one of antivenom pharmacokinetics, whereby different types of antivenoms (ranging from intact immunoglobulin G to Fab fragments) have differential rates of penetration into the deep tissues and also dramatically varying rates of clearance from the body [213,214,215,216,217]. The rapid clearance rate of Fab fragment antivenoms has led to clinical issues such as recurring coagulopathy which in some cases has necessitated continuous intravenous infusion in Fab fragment antivenoms such as CroFab [218,219,220,221,222,223]. Further complicating matters is that venoms rich in low molecular weight toxins will be able to penetrate deeper into the tissues than antivenoms consisting of large molecular weight molecules such as intact IgG. Thus, there are several areas of controversy regarding antivenom dosage due to these compounding variables with regard to how much to give initially, how often to give more (and what amounts), and how long to continue administration.

Due to concerns about adverse reactions to antivenom, some doctors are reticent to give antivenom despite the risk of permanent injury to the patient from untreated envenomation in addition to the patient suffering during the acute phase of envenomation [224,225,226]. Indeed, it has been shown that use of antivenom is linked to better limb recovery, even for typically sublethal envenomations such as by *Agkistrodon contortrix* [227]. The attitude about withholding antivenom due to allergy concerns has no doubt been shaped by the historically higher allergenicity of previous generation antivenoms (e.g., Wyeth antivenom for pitviper envenomations in North America) versus the lower allergenicity of more recent products such as CroFab (also for pitviper envenomations in North America) [228,229]. 

#### 4.3.4. Antivenom Cross-Reactivity

Aside from the above dosage issues are the considerations as to what evidence supports the administration of a particular antivenom to treat envenomation by a species not used in the immunizing mixture. Antivenom cross-reactivity poses a complex biochemical challenge as even single amino acid substitutions in toxin molecules may be enough to interfere with recognition by the antibody [230,231]. In clades such as sea snakes, there is a remarkable level of cross-reactivity due to the extreme conservation of diet (fish in this case) and the consequent streamlining of the venom profiles [232,233]. This level of cross-reactivity was fortuitous since it transpired that the antivenom production was done using a completely different species (*Hydrophis* [*Enhydrina*] *schistosa*) than the target species (*Hydrophis* [*Enhydrina*] *zweifeli*) [234]. This error occurred because the Australian antivenom manufacturer deemed it more economical to collect venom from Malaysia than from Australia from what was at that time considered to be simply a different population of the same species. However, later taxonomic work showed that the populations were two different sea snake species which last shared a common sea snake ancestor over seven million years ago [235]. Instead, of being the same species, they instead represent a remarkable case of morphological convergence, the first documented for any venomous snake [234]. Other cases of intergeneric cross-reactivity include the Australian elapid clade comprising *Hoplocephalus*, *Notechis*, *Paroplocephalus* and *Tropidechis* [236]. In this case, the procoagulant toxins (a mutated form of blood factor Xa) were extraordinarily similar between the venoms [237] and did not display the accelerated molecular evolution seen in other toxin types such as three-finger toxins (3FTx) [237,238,239]. The unusual conservatism of factor Xa toxins is presumably due to the target (prothrombin) being under tight endogenous regulatory control and thus likely evolving under stabilizing selection. Consequently, in this clade the available antivenom performed extremely well against all species despite their separation by up to eight million years and the antivenom produced using only venom from *Notechis scutatus* [237].

However, in other lineages, even closely-related species or regional variations within a species may be poorly neutralized. *Echis* species, for example, are particularly problematic in this regard. In Africa, the unscrupulous marketing of Indian antivenoms has led to a dramatic increase in snakebite deaths from *Echis* species [240]. These antivenoms are marketed without any data to support their efficacy and independent testing revealed their failure to neutralize African *Echis* venoms [241,242,243]. Indian antivenoms are also marketed closer to home, in nearby Pakistan, but studies have shown poor performance against *Bungarus* and *Naja* in Pakistan [244]. Examples of variable cross-reactivity between subspecies of venomous snake include *Crotalus scutulatus* [245] and between congeneric species including *Micrurus* [246,247] and *Bothrops* [248,249,250,251,252,253]. Some medically important genera remain without any effective antivenom including colubrids such as *Thelotornis* [254] and lamprophiids such as *Atractaspis* [255].

#### 4.3.5. In Vitro Methods for Ascertaining Antivenom Effectiveness

Antivenomic methods which measure binding in the absence of a functional test (cf. [256]) must be interpreted with particular caution as binding does not equate with neutralization. In some cases, binding has been shown to correlate with inhibition of function [48,230,231,257] but binding alone cannot always be assumed to inhibit function. Thus, there is a need to correlate binding and in vivo neutralization. Such considerations are clinically important as some studies that measure bound venom in the plasma of human snakebite victims have equating binding as being and thus made antivenom dosage recommendations based upon this untested relationship [206,209,258]. Indeed, the absence of correlation studies has been noted by critics of this approach [211,212] (see Section 4.3.3). 

Methods to ascertain inhibition of function typically involve preincubation of venom and antivenom followed by functional assaying. This is an effective way of showing when an antivenom does not work since if it does not impede activity under such idealized circumstances, then the likelihood of effective neutralization in the more dynamic in vivo clinical setting is remote. The difficulty lies extrapolating positive results into real world applications. Incubation time is a significant variable since prolonged incubation allows for slower, weaker interactions to occur that may not occur during the shorter encounters in vivo. The current World Health Organization antivenom testing protocol calls for a 30 min incubation of antivenom with venom [207], with this protocol based upon methods largely unchanged since the 1950s [259,260,261]. Long versus short incubation times resulted in very different taxonomical patterns of antivenom cross-reactivity in studies of *Echis* venoms; investigation of the Echi-Tab-ICP antivenom using a 30 min incubation time [262] ascertained this antivenom as having a greater span of cross-reactivity than later work which used 2 min incubation [242]. The latter method selects for high-affinity, rapid binding as this better reflects the interactions in the fast moving and dynamic in vivo system. In addition, it has been shown that venom enzymes may autocatalyze and lose activity during longer incubation periods [164], which would also skew venom potency and antivenom efficacy interpretations in protocols using longer incubation times. 

It has been shown that calcium and phospholipid cofactors can have a very large influence on coagulotoxic enzymatic function [236,242,254,255]. Mechanisms of ascertaining enzymatic function, and the antivenom inhibition thereof, must always include these cofactors. While some studies have indeed included both [236,242,254,255,263,264,265] some only include calcium [164,266,267,268], and other studies included neither cofactor [269,270,271,272]. Therefore, omission of one or both cofactors can potentially skew results and thus the interpretation not only of relative toxicity but also of antivenom efficacy. Thus the inclusion of calcium and phospholipid cofactors is absolutely essential when constructing the study design. Their omission can lead to the mistaken impression that some lineages have reduced or even entirely lacking procoagulant function, such as has occurred in [272].

## 5. Conclusions

This review illustrates how the inherent multifactorial nature of snakebite results in it being not just a medical issue but equally an economic issue as well as a social issue. Thus, recommendations arising from this review not only include innovative lines of research and improvements in clinical training but also the involvement of economists and sociologists. Particular areas of contention in the absence of conclusive evidence urgently requiring further research include the use of pressure-bandages and whether simple measurements of antivenom binding are reliable indicators of the neutralization capacity of the antivenom and thus whether such measurements are appropriate for formulating clinical guidelines in regards to antivenom dosage. Similarly, sociological research must be undertaken to find ways of bringing about the cultural shifts so that the involvement of traditional healers and their ineffective remedies in snakebite is phased out. Such shifts are only feasible if antivenom is locally available and thus economists must be engaged to facilitate reliable supply chains and distributions networks. It is only by simultaneously tackling multiple, seemingly unrelated, aspects of snakebite that profound improvements may occur in this most neglected of all tropical diseases.
